# Novel Viruses Isolated from Mosquitoes in Pantanal, Brazil

**DOI:** 10.1128/genomeA.01195-16

**Published:** 2016-11-03

**Authors:** Alex Pauvolid-Corrêa, Owen Solberg, Dinair Couto-Lima, Rita Maria Nogueira, Stanley Langevin, Nicholas Komar

**Affiliations:** a Division of Vector-Borne Diseases, Centers for Disease Control and Prevention (CDC), Fort Collins, Colorado, USA; b Systems Biology Department, Sandia National Laboratories, Livermore, California, USA; c Laboratório de Flavivírus, Instituto Oswaldo Cruz, Fundação Oswaldo Cruz (Fiocruz), Ministério da Saúde, Rio de Janeiro, Brazil

## Abstract

Genomic sequences are described from five novel viruses and divergent strains of Brejeira and Guaico Culex viruses from mosquitoes collected in Pantanal, Brazil, in 2010.

## GENOME ANNOUNCEMENT

We describe here the genomes of five novel viruses and divergent strains of Brejeira (BRJV) and Guaico Culex (GCXV) viruses isolated from pooled mosquitoes captured with CDC light traps from Pantanal, Mato Grosso do Sul state, in west-central Brazil, in April 2010. Genomic sequences were derived from mosquito cell cultures by Illumina technology (1–3) (Table 1).

**TABLE 1:** Genomic sequences identified from 12 isolates of 7 viruses isolated from mosquitoes in Pantanal, Brazil, in April 2010^a^

GenBank accession no.	Virus name	Segment	Virus family	Pool no.	Mosquito species (no. of specimens)
KT966481	Kaiowá virus		Unclassified	313	*Culex* (*Culex*) spp. (15)
KT966482	Brejeira virus		Unclassified	313	*Culex* (*Culex*) spp. (15)
KT966483	Brejeira virus		Unclassified	324A	*Culex* (*Culex*) spp. (27)
KT966484	Guató virus		Unclassified	324A	*Culex* (*Culex*) spp. (27)
KT966485	Brejeira virus		Unclassified	324B	*Culex* (*Culex*) spp. (23)
KT966486	Guató virus		Unclassified	324B	*Culex* (*Culex*) spp. (23)
KT966487	Brejeira virus		Unclassified	332A	*Culex* (*Culex*) spp. (26)
KT966490	Guató virus		Unclassified	332A	*Culex* (*Culex*) spp. (26)
KT966491	Ofaié virus		*Mesoniviridae*-like	360	*Mansonia* spp. (25)
KT966492	Terena virus	S	*Bunyaviridae*-like	360	*Mansonia* spp. (25)
KT966493	Terena virus	M	*Bunyaviridae*-like	360	*Mansonia* spp. (25)
KT966494	Terena virus	L	*Bunyaviridae*-like	360	*Mansonia* spp. (25)
KT966495	Kadiwéu virus		*Mesoniviridae*-like	407	*Culex* (*Culex*) spp. (50)
KT966498	Guaico Culex virus	1	Unclassified	407	*Culex* (*Culex*) spp. (50)
KT966499	Guaico Culex virus	2	Unclassified	407	*Culex* (*Culex*) spp. (50)
KT966500	Guaico Culex virus	3	Unclassified	407	*Culex* (*Culex*) spp. (50)
KT966501	Guaico Culex virus	4	Unclassified	407	*Culex* (*Culex*) spp. (50)
KX762047	Guaico Culex virus	5	Unclassified	407	*Culex* (*Culex*) spp. (50)

^a^ The taxonomy, source, and relationship to similar known viruses were determined by nucleotide BLAST (“BLASTx” in GenBank).

Four BRJV isolates from *Culex* (*Culex*) spp. yielded 99% identity among themselves, but 88% to 90% identity with a BRJV isolated in northern Brazil, indicating the detection of a divergent strain of this recently described *Negevirus*-like virus (4).

We report here the first Brazilian detections of GCXV, a recently described multicomponent Jingmenvirus (5). The five genomic segments from *Culex* (*Culex*) spp. pool number 407 aligned with coverages from 78% to 87% and identities from 97% to 100% with published sequences. A second isolate from pool number 313, also *Culex* (*Culex*) spp., was the same strain, based on partial sequences (data not shown). In Brazil, the first detection of a Jingmenvirus was from tick collections in southeast Brazil, and called Mogiana tick virus (6).

Five novel viruses were detected and named to honor indigenous tribes of Mato Grosso do Sul state (Terena, Ofaié, Kadiwéu, Kaiowá, and Guató viruses). Translated nucleotide identities ranged between 32% and 73% to known viruses. The following abbreviations are suggested for the novel viruses: TERV, OFAV, KADV, KAIV, and GUTV.

TERV isolated from a *Mansonia* spp. pool aligns with viruses of a newly described genus within *Bunyaviridae* called *Phasmavirus* (7). It contains the standard three-segmented genome with the small (S), medium (M), and large (L) segments of other bunyaviruses.

OFAV derived from the same mosquito pool as TERV. OFAV has a large genome of 19.8 kb and most closely aligns (<66% predicted amino acid sequence identity) with Méno virus, recently described within the family *Mesoniviridae* (8). KADV is also related to Méno virus (<71% identical) and is similarly large (20.4 kb). OFAV and KADV align with each other with 70% nucleotide identity over a query coverage of 68%.

GUTV and KAIV isolated from *Culex* (*Culex*) spp. most closely align with a recently described virus from China called Wuchang Cockroach virus 3. The three isolates of GUTV are 99% identical to each other, whereas KAIV and GUTV are 30% divergent at the nucleotide level. These viruses consistently accompanied isolates of BRJV. Whether the accompaniment of these viruses is coincidence, or they derived from coinfected mosquitoes within each pool, is unknown. However, KAIV and GUTV genomes could in fact be independent genomic segments within a multisegmented genome that includes BRJV.

To evaluate the host range of these virus isolates, attempts to generate cytopathic effect (CPE) were made with human, monkey, frog, bat, mosquito, and tick cell cultures. A clear CPE was observed only in mosquito cells. However, the capacity of these novel viruses to cause CPE in vertebrate and invertebrate cells was not fully evaluated.

Pantanal’s biodiversity has recently produced other mosquito-borne viruses, and may be useful for studying virus evolution (9–14).

### Accession number(s).

The sequences have been deposited in GenBank under the accession numbers listed in Table 1.
